# *In Vivo* Antiplasmodial and Analgesic Effect of Crude Ethanol Extract of *Piper guineense* Leaf Extract in *Albino* Mice

**DOI:** 10.1155/2016/8687313

**Published:** 2016-06-30

**Authors:** A. Y. Kabiru, G. F. Ibikunle, D. A. Innalegwu, B. M. Bola, F. M. Madaki

**Affiliations:** ^1^Department of Biochemistry, Federal University of Technology, PMB 65, Minna, Niger State, Nigeria; ^2^Department of Chemistry, Federal University of Technology, PMB 65, Minna, Niger State, Nigeria; ^3^Centre for Genetic Engineering and Biotechnology, Global Institute for Bio-Exploration Unit, Federal University of Technology, PMB 65, Minna, Niger State, Nigeria

## Abstract

Antiplasmodial and analgesic effects of crude ethanol extract of* Piper guineense* was investigated in mice. The antiplasmodial and analgesic efficacy of the extract was judged on its ability to reduce parasitemia and writhing, respectively, in mice. The antiplasmodial screening involved treating infected mice with 200, 400, and 600 mg/kg body weight of extract while the positive control group was given standard artesunate drug. The analgesic test was carried out by administering 1000, 1500, and 2000 mg/kg body weight of extract to three groups of healthy mice, respectively, after induction of pain with 0.75% acetic acid. The positive control group was given aspirin drug. Parasitemia was reduced by 28.36%, 43.28%, and 62.69% in a dose-dependent pattern in the curative test which was significantly different (*P* < 0.05) from 96.03% of the standard drug. The reduction of writhing by mice given the extract was also dose-dependent (36.29, 45.43, and 59.07%). Aspirin drug was however more effective (86.36%). The extract was safe at 2000 mg/kg body weight. Phytochemical screening revealed the presence of flavonoids, tannins, phlobatannins, terpenoids, and coumarins. Result obtained in this study demonstrated the efficacy of ethanol extract of* Piper guineense* as an antiplasmodial and analgesic agent.

## 1. Introduction

Malaria inflicts a heavy toll on the health and quality of life of the residents of intertropical countries, affecting one-third of the world's human population [1, 2]. Communities in Africa bear the brunt of this parasitic, blood-borne disease, with an estimated 90% of all malarial deaths occurring in African children below the age of 5 years [3]. Previous malaria control efforts relied heavily on chloroquine as a cheap and effective treatment.* Plasmodium falciparum* resistance to chloroquine emerged in the 1960s and rendered the drug ineffective by the early 1990s, contributing to a substantial rebound in malaria mortality [4]. These parasites have developed at least partial resistance to nearly every antimalarial remedy introduced to date including the sulfadoxine-pyrimethamine combination drug [5].

Currently, one of the most prescribed antimalarial drugs against* Plasmodium falciparum* parasite infection is Artemisinin, a compound derived from Chinese worm wood trees which is significantly more expensive to produce than older, synthetic forms of malaria medicine [6].

In 2005, the World Health Organization (WHO) began recommending the use of Artemisinin-based combination therapies (ACTs) as the preferred first-line antimalarial drug. These pair an extremely potent but short-lived Artemisinin derivative (typically Artesunate or artemether) with a longer-acting antimalarial drug such as lumefantrine, amodiaquine, or mefloquine.

There has been an aggressive search for plant-based drugs due to resistance of* Plasmodium* species to presently used antimalarial drugs. The antiplasmodial activity of* Piper guineense* seed extract was reported by Gbadamosi et al. [7]. The present study was designed to screen the antiplasmodial activity of the leave extract of* Piper guineense* using ethanol as solvent of extraction.

*Piper guineense* is a West African species of pepper; the spice derived from its dried fruit is known as West African pepper, Ashanti pepper, Benin pepper, False cubeb, Guinea cubeb, Uziza pepper, or guinea pepper and called locally Kale, Kukauabe, Masoro, Sasema, and Sorowisa [8].

*Piper guineense* is a perennial woody climber that grows up to 10 m or more in height. Its leaves are alternate and simple with a petiole 2–5 cm long. It is a plant of the wet tropics that requires heavy and well distributed rainfall and temperature. The ideal soil for production is a well-drained alluvium with high humus content. It can also be grown in red laterite soil. The leaves of* Piper guineense* are used as a leafy vegetable pepper in most of the African soups. The leaves and fruits are also used as flavor in most dishes. Traditionally, people believe that they have medicinal properties.* Piper guineense* is added to food meant for pregnant and nursing mothers as a medicinal spice and among the postpartum women. It is claimed that it assists in the contraction of the uterus [9].

In Nigeria and other developing nations of the world, especially in Africa, many low income earners and residents of remote villages rely almost exclusively on traditional medicines for treatment of several diseases, including malaria, convulsion, epilepsy, infertility, and dysentery. Extracts from barks, leaves, roots, or seed of plants (soaked in local gin) are used in the preparation of syrups or other medicinal formulations. Medicinal plants often used in such preparations include* Piper guineense* seeds usually given to nursing mothers, for the normalization of the womb after delivery [9].

The seeds, leaves, and sometimes the stems are used in preparing soup. It imparts “heat” and a spicy pungent aroma to food. The medicinal properties of* Piper guineense* exert bacteriostatic and bactericidal effects on some bacteria. The leaves are considered aperient, carminative, and eupeptic. They are also used for the treatment of cough, bronchitis, intestinal disease, and rheumatism. The leaves are also used for management of female infertility while the fruits are used as an aphrodisiac [8].

## 2. Materials and Methods

### 2.1. Materials

#### 2.1.1. Plant Materials

The fresh leaves of* Piper guineense* were obtained from Oboloafor village in Enugu State, Nigeria, in the month of November, 2013. The plant was identified by a botanist in the Department of Biological Sciences, Federal University of Technology, Niger State. A specimen was deposited in the herbarium section of the department. The leaves were washed and dried for 10 days at room temperature in the Department of Biochemistry laboratory, Federal University of Technology, Niger State.

#### 2.1.2. Experimental Animals

Thirty-six healthy albino mice, weighing between 20 and 25 g, were obtained from the Animal House of Department of Zoology, National Institute of Trypanosomiasis Research (NITR), Kaduna. The mice were housed in a well-ventilated laboratory cage and allowed free access to clean water and food to allow acclimatization for two weeks in the department laboratory.

#### 2.1.3. Plasmodium Parasite

The* Plasmodium* species,* Plasmodium berghei,* was obtained from National Institute for Pharmaceutical Research and Development (NIPRID), Idu, Abuja, Nigeria. The parasite was subsequently maintained in mice by continuous passaging into healthy mice.

### 2.2. Methods

#### 2.2.1. Sample Preparation

Fresh leaves of* Piper guineense* were washed with clean water to remove dust and sand. The leaves were air-dried at room temperature to constant weight. The dried leaves were then blended using a blender to powdered form.

#### 2.2.2. Extraction Procedure

Fifty grams (50 g) of the powdered sample was weighed into a round bottom flask and extracted with 400 mL of absolute ethanol at 60°C for 2 hours by reflux method according to the method described by Ugwu et al. [10] and Kabiru et al. [11]. The extract was filtered using muslin cloth and the filtrate was slowly evaporated to dryness using the steam bath. The crude extract was weighed and yield kept in the freezer until being required for use.

#### 2.2.3. Preliminary Phytochemical Analysis

The phytochemical screening of* Piper guineense* leaf extract was carried out using the method of Trease and Evans, 1989 [12], to detect the presence of alkaloids, saponins, glycosides, flavonoids, tannins, steroids, terpenoids, anthraquinones, phlobatannin, coumarins, and volatile oils.

#### 2.2.4. Analgesic Test

Analgesia was accessed by the method of Mishra et al. [13]. Fifteen (15) mice of average weight 25 g comprising both sexes were divided into 5 groups (A–E) consisting of 3 mice each. Groups A, B, and C were given 1000, 1500, and 2000 mg kg^−1^ body weight, respectively, of the plant extract intraperitoneally (i.p), 60 minutes before acetic acid (0.75% v/v) was given through the same route. Parallel tests were carried out using acetylsalicylic acid (ASA 150 mg/kg body weight per day i.p.) as reference (group D) and normal saline (20 mL/kg body weight per day i.p.) as control (group E). Five minutes was allowed to elapse before the amount of writhing by mice at intervals of 10 minutes for a period of 60 minutes was counted using a tally counter.

#### 2.2.5. Parasite Inoculation

The method described by Kabiru et al. [11] was used for the inoculation of parasites into the experimental animals. Each mouse was inoculated on day zero, intraperitoneally with 0.2 mL of parasite infected blood containing approximately 1 × 10^7^ *Plasmodium berghei/*parasitized red blood cells. In addition, the newly inoculated animals were monitored daily to determine expression of parasites in circulation.

#### 2.2.6. Antiplasmodial Test

the method described by Souleymane et al. [14], was used to screen for antiplasmodial property of the extract. Fifteen (15) mice of average weight 25 g consisting of both sexes were inoculated with 0.2 mL of diluted parasitized blood obtained 72 hours prior to the test from a highly parasitized mice with average number of parasites of 10^3^ parasites/mL of blood. They were divided into 5 groups (A–E) consisting of 3 mice each. Groups A, B, and C were given a daily dose of 200, 400, and 600 mg/kg^−1^ body weight, respectively, of the plant extract intraperitoneally while group D was treated with 50 mg kg^−1^ body weight of the standard drug, Artesunate, as the positive control group and group E was left untreated as the negative control group. On the fifth day of the treatment, blood was collected from the tail of each mouse and a thin film was made on the slide, fixed with methanol, and stained with Giemsa stain so that the average percentage parasitaemia could be evaluated for each of the doses using the formula below. Any death that occurred during this period was noted and used to determine the mean survival time [15].

#### 2.2.7. Estimation of Parasitaemia in Curative Test

Parasitaemia was monitored in blood obtained from the tail of the mouse. Thin smear of the blood film was prepared on a microscope slide. The film was allowed to dry for about 3–5 minutes, fixed with methanol, and stained with a diluted Giemsa at pH 7.2 for 30 minutes. The stained slide is washed under a running tap and allowed to dry before viewing under the microscope at ×100 magnification to access the level of the parasitaemia. The percentage parasitaemia was calculated using the following formula:

(1) $$\begin{array}{c} \text{percentage parasitaemia}=\frac{Pc-Pt}{Pc}\times 100, \end{array}$$

where Pc is the parasitaemia in the control group and Pt is the parasitaemia in the treated group.

The thin blood film was methanol-fixed and stained with diluted Giemsa stain using buffered water at pH 7.2 to emphasize the parasite inclusion in the red blood cells.

#### 2.2.8. Estimation of Packed Cell Volume (PCV)

Blood sample was collected from the tail of each mouse with a capillary tube by capillary action. The tube was sealed with plasticine at one end. The sealed capillary tubes were then arranged on the hematocrit centrifuge and set to spin at 1200 revolutions per minute for five minutes. The PCV was read on the hematocrit reader and recorded.

#### 2.2.9. Data Analysis

Data obtained in this study were analyzed using the statistical software SPSS 16.0, 2007 version (SPSS Inc., Chicago, Illinois, USA). Numerical data were presented as mean ± standard error of mean (SEM). The significance of the mean difference between two independent groups was determined using Duncan and one-way analysis of variance (ANOVA) while multiple comparisons were used when comparing more than two groups. A *P* value < 0.05 was considered.

## 3. Results

### 3.1. Percentage Yield of Crude Ethanolic Extract of* Piper guineense* Leaf Extract

the crude ethanol extract of* Piper guineense* leaf extract yielded 27.24% w/w which was kept in the freezer until being required for use.

### 3.2. Phytochemical Composition of Crude Ethanolic Extract of* Piper guineense* Leaf Extract

A preliminary phytochemical screening by the method of Trease and Evans [12] pointed towards the presence of flavonoids, tannins, anthraquinones, steroids, phlobatannins, coumarins, and proteins while saponins, alkaloids, and volatile oils were not detected.

### 3.3. Acute Toxicity of Crude Ethanol Extract of* Piper guineense* Leaf in Mice

Acute toxicity studies of crude ethanol extract of* Piper guineense* leaves in mice showed that the extract was safe at a dose of 2000 mg/kg body weight with no mortality recorded. However, loss of appetite and weakness were observed in the animals for about an hour on administration of the extract. Breathing, eye color, and furs position were normal.

### 3.4. Analgesic Effect of Crude Ethanol Extract of* Piper guineense* Leaf in Mice

**Figure 1:**
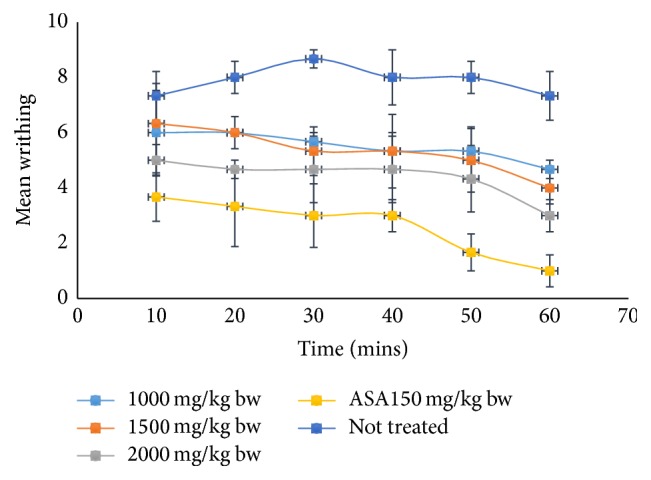
Analgesic effect of crude ethanol extract of* Piper guineense* leaf in mice.

Figure 1 shows the analgesic effect of crude ethanolic extract of* Piper guineense* leaf in mice at a concentration of 1000, 1500, and 2000 mg kg^−1^ body weight with acetylsalicylic acid (ASA) of concentration 150 mg kg^−1^ body weight as the standard or the positive control. The extract reduced the writhing of the mice by 36.29%, 45.43%, and 59.07% at a dose of 1000, 1500, and 2000 mg kg^−1^ body weight, respectively, which was significantly different (*P* < 0.05) when compared to the standard drug, Aspirin, which reduced the writhing by 86.36%.

### 3.5. Antiplasmodial Activity of Crude Ethanol Extract of* Piper guineense* Leaf on* Plasmodium berghei*-Infected Mice

**Figure 2:**
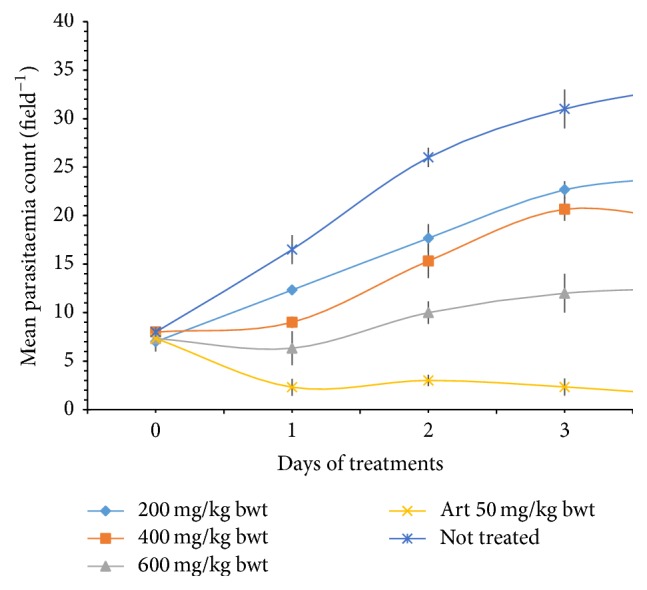
Antiplasmodial activity of crude ethanolic extract of* Piper guineense* leaf in* Plasmodium berghei*-infected mice.

Figure 2 shows the average parasitaemia in the blood of mice infected with* Plasmodium berghei* and treated intraperitoneally with crude ethanol extract of* P. guineense* leaf of concentration of 200, 400, and 600 mg kg^−1^ body weight per day and the standard drug Artesunate at a dose of 50 mg kg^−1^ (Art 50 mg kg^−1^). The screening was carried out for a period of five days with parasitaemia count recorded daily. The ethanol extract at a dose of 200, 400, and 600 mg/kg body weight per day reduced the level of parasitaemia by 28.36%, 43.28%, and 62.69%, respectively, after treating for five days in the curative test. This was significantly lower (*P* < 0.05) when compared to the standard drug, Artesunate, which reduced the parasitaemia by 96.03%. Based on the 62.69% obtained with 600 mg/kg body weight, there is every possibility that higher doses of the extract will produce better parasite clearance.

### 3.6. Effect of Crude Ethanol Extract of* P. guineense* Leaf on the Mean Body Weight of Mice of* Plasmodium berghei*-Infected Mice

**Figure 3:**
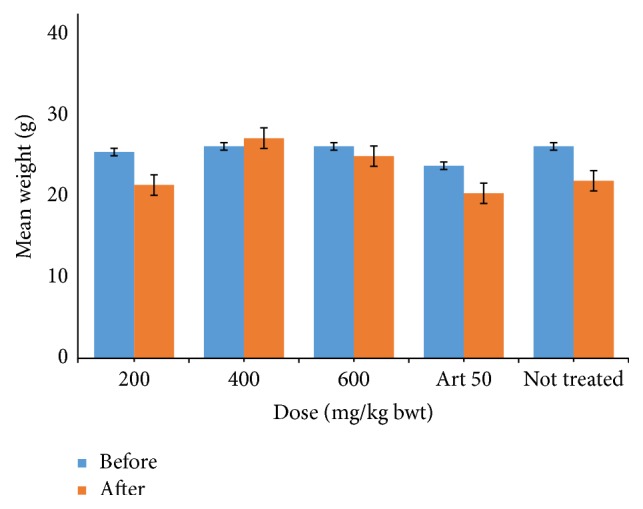
Effect of crude ethanol extract of* P. guineense* leaf on the mean body weight of* Plasmodium berghei*-infected mice.

From the result of the curative test (Figure 3) carried out on the mice, there was no significant change in the body weight of the mice before and after inoculation in each of the respective doses.

### 3.7. Effect of Crude Ethanol Extract of* P. guineense* Leaf on the Pack Cell Volume (PCV) of* P. berghei*-Infected Mice

The packed cell volume generally decreased after infection and was not reversed after treatment with the extract and thus there was significant difference (*P* < 0.05) in PCV before and after treatment with extract, while the situation was different for the group treated with standard drug, Artesunate. The initial decrease in PCV was reversed and there was no significant difference between initial PCV and final PCV of the standard drug-treated group.

### 3.8. Effect of Crude Ethanol Extract of* P. guineense* Leaf on the Mean Survival Time of* P. berghei-*Infected Mice

The mean survival time of the mice treated with the extract at different doses indicates that the antiplasmodial activity was dose-dependent, because the mice treated with the highest dose (600 mg kg^−1^ body weight) survived longer than other groups. However, mice treated with Artesunate survived for about 30 days, while mice in the negative control group died within 3 days after infection.

## 4. Discussion

The potency of medicinal plants depends solely on their active phytochemical components which produces a definite physiological action on the human body and is responsible for their numerous bioactivities [8]. The preliminary phytochemical composition of crude ethanolic extract of* Piper guineense* leaf pointed towards the presence of flavonoids, tannins, anthraquinones, steroids, phlobatannins, coumarins, proteins, cardiac glycoside, and terpenoids. The chosen method, however, does not allow the unambiguous identification of such natural products so that further detailed phytochemical studies will have to be carried out.

The acute toxicity screening of the plant extract also indicates the extract to be safe in the mice in compliance with the Organization for Economic Cooperation and Development (OECD) guidance document on acute toxicity (2000). However, loss of appetite and weakness were observed in the animals in the first hour of administration of the extract at various doses but breathing, eye color, skin, and furs position were normal throughout the study period.

**Figure 4:**
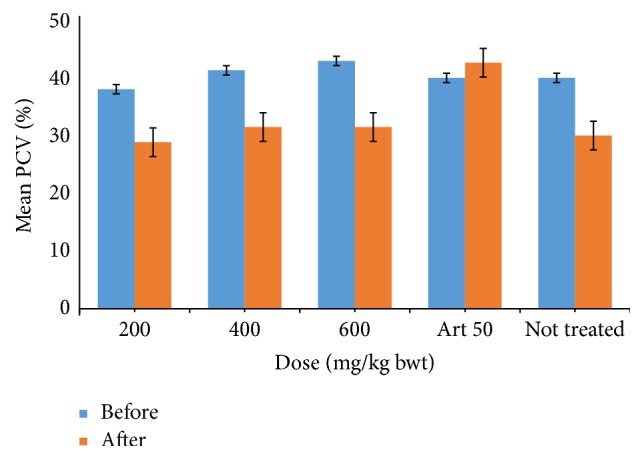
Effect of crude ethanol extract of* P. guineense* leaf on the pack cell volume (PCV) of* P. berghei*-infected mice.

The crude ethanol extract reduced the level of parasitaemia in a dose-dependent manner with the highest dose reducing the parasitaemia by 62.69% after treatment for five days in the curative test. This was significantly different (*P* < 0.05) when compared to the standard drug, Artesunate, which reduced the parasitaemia by 96.03% (Figure 2). Phytochemical compounds such as terpenoids and flavonoids are thought to be responsible for antiprotozoal and antiplasmodial activity of most plants such as Artemisinin, the main active component of the traditional Chinese antimalarial plant (Qinghaosu) [16–19]. The packed cell volume (PCV) generally decreased after infection because anaemia is a common problem that is associated with malaria (Figure 3). Also, there was no significant change in the body weight of the mice at all doses (Figure 4), implying that other than direct parasiticidal effects, the plant may possess other pharmacological benefits to the hosts.

**Figure 5:**
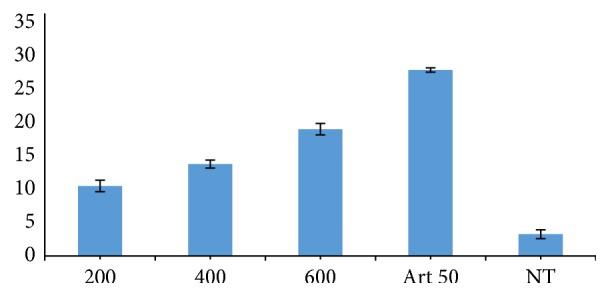
Mean survival time of* P. berghei-infected* mice. NT: not treated.

The mean survival time for the mice treated with the extract at all doses used indicated that the antiplasmodial activity of the extract was dose-dependent, because the group treated with the highest dose survived longer (20 days) than other groups. However, the group treated with Artemisinin survived for about 30 days, while mice in the negative control group died three days after infection (Figure 5). There is an indication in this result that a higher dose of extract could produce a longer survival time.

In the analgesic study, the extract was found to cause a reduction in writhing of the mice when 1000, 1500, and 2000 mg kg^−1^ body weight doses were administered after induction of pain. The result also shows that the extract is dose-dependent with the highest dose reducing the writhing by 59.07% in 60 minutes of the reaction time. This was significantly different (*P* < 0.05) when compared to the standard drug, acetylsalicylic acid, which reduced the writhing by 86.36% (Figure 1). Flavonoid and terpenoids as revealed by the phytochemical composition are thought to be attributed to this activity as flavonoid and terpenoids are known to be used as analgesic agent in modern medicine [20].

The analgesic activity of* P. guineense* further justifies its suitability as an antimalarial agent. This is because species with complimentary pharmacological effects are better in the resolution of* Plasmodium* infection and its clinical manifestations than plants which only clear parasites from circulation [21].

## 5. Conclusion

It is convenient to conclude from the results obtained in this study that the ethanol extract of* Piper guineense* possesses phytochemicals with antiplasmodial and analgesic potentials capable of being packaged as ethnomedicine.
